# Shared and distinct alterations in brain connectivity and cognitive function in subthreshold and major depression

**DOI:** 10.1017/S0033291725102687

**Published:** 2026-03-09

**Authors:** Shuming Zhong, Chao Chen, Pan Chen, Xinyue Tang, Guanmao Chen, Shunkai Lai, Yiliang Zhang, Wenhao Ma, Yuan Zhang, Shu Zhang, Zhangzhang Qi, Qian Tao, Yanbin Jia, Ying Wang

**Affiliations:** 1 Department of Psychiatry, First Affiliated Hospital, Jinan University, Guangzhou 510630, China; 2 Medical Imaging Center, First Affiliated Hospital, Jinan University, Guangzhou 510630, China; 3 Department of Public Health and Preventive Medicine, School of Basic Medicine, Jinan University, Guangzhou 510632, China

**Keywords:** brain network, cognitive function, functional connectivity, major depressive disorder, subthreshold depression

## Abstract

**Background:**

Subthreshold depression (StD) is considered a prodromal stage of major depressive disorder (MDD). This study aims to investigate the neurobiological mechanisms of StD by analyzing functional connectivity (FC) and cognitive function in comparison to MDD.

**Methods:**

A total of 153 StD individuals, 188 MDD patients, and 110 healthy controls (HCs) were studied using resting-state functional magnetic resonance imaging (fMRI). Whole-brain FC was calculated using seeds from the default mode network (DMN), salience network (SN), executive control network, and affective network (AN). Cognitive function was assessed across seven domains.

**Results:**

StD showed only a deficit in social cognition, while MDD exhibited multidomain cognitive impairments compared to HCs. Both MDD and StD exhibited reduced FC between the right anterior insula (AI) and the left inferior frontal gyrus (IFG), and increased FC between the right subcallosal cingulate cortex and the left posterior cingulate cortex (PCC), key areas of the SN and AN, compared to HCs. MDD particularly showed decreased connectivity between the left PCC and the left middle temporal gyrus, and within the left PCC, while no abnormal FC of the DMN was found in StD. Altered AI-IFG FC was positively correlated with social cognition in StD.

**Conclusions:**

Abnormal connectivity patterns of the SN and AN may contribute to the development of depressive symptoms in StD and MDD, while altered FC of the DMN may be involved in the onset of the disease. A social cognition deficit appeared first in StD, relating to the abnormal connectivity of the SN.

## Introduction

Subthreshold depression (StD), also known as subsyndromal or subclinical depression, is characterized by the presence of at least one core symptom and two to four additional depressive symptoms persisting for a minimum duration of 2 weeks, without meeting the full diagnostic criteria for major depressive disorder (MDD) (Rodriguez, Nuevo, Chatterji, & Ayuso-Mateos, 2012). Individuals experiencing StD exhibit greater similarities to those diagnosed with MDD than to individuals without any depressive symptoms (Noyes et al., 2022), and they possess an increased risk of progressing to a depressive disorder (Hermens et al., 2004). StD is considered a prodromal phase of MDD, substantially heightening the likelihood of developing full-blown MDD (Lee et al., 2019). Furthermore, StD is linked to a reduced quality of life and financial difficulties (Cuijpers et al., 2007), and it often co-occurs with anxiety symptoms, thereby imposing considerable burdens on society, families, and individuals (Racine et al., 2021). Early intervention or targeted prevention strategies, such as psychological interventions for StD, have the potential to prevent the onset of MDD (Cuijpers et al., 2014). However, the diagnosis of StD predominantly depends on clinical evaluations and the expertise of psychiatrists (Cuijpers, Quero, Dowrick, & Arroll, 2019), which heightens the risk of misdiagnosis and inappropriate treatment. Therefore, it is crucial to elucidate the neurobiological mechanisms underlying StD to enable accurate and timely differentiation between individuals with StD and healthy individuals.

Functional magnetic resonance imaging (fMRI) has been extensively utilized in neuroimaging studies of StD and MDD. Current evidence indicates that depressive symptoms may arise from disruptions in specific brain networks rather than isolated brain regions (Wang, Hermens, Hickie, & Lagopoulos, 2012). Previous research has indicated that abnormal patterns of brain connectivity are increasingly implicated in the pathogenesis of StD (Dedovic et al., 2016; Gao et al., 2016; Philippi, Motzkin, Pujara, & Koenigs, 2015). Over the past decade, research within the network framework has consistently implicated four specific networks in the pathophysiology of depression: the default mode network (DMN), the salience network (SN), the executive control network (ECN), and the affective (limbic) network (Kaiser, Andrews-Hanna, Wager, & Pizzagalli, 2015). Research on MDD has predominantly focused on the DMN, particularly the posterior cingulate cortex (PCC) and middle temporal gyrus (MTG), which are implicated in rumination (Hamilton, Farmer, Fogelman, & Gotlib, 2015). However, studies have reported both increased (Greicius et al., 2007; Zhu et al., 2012) and decreased functional connectivity (FC) within the DMN in individuals with MDD (Guo et al., 2014). Furthermore, a positive correlation has been identified between the severity of depression and enhanced FC from the subgenual anterior cingulate cortex (ACC) to other regions within the DMN (Berman et al., 2011; Greicius et al., 2007). The SN, similar to the ventral attention network, has also been consistently associated with the pathophysiology of MDD (Kaiser et al., 2015). The SN, primarily comprising the insula and the ACC, is crucial for detecting salient interoceptive and external changes, managing neural responses to significant stimuli, and facilitating transitions between ECN- and DMN-dominant activity (Sridharan, Levitin, & Menon, 2008). Additionally, dysfunctional regulation of negative emotions within the AN may represent a significant characteristic of MDD (X. Zhang et al., 2014). StD has been associated with altered FC between ACC subregions, increased habenula FC within the DMN, and decreased FC within the SN (Ely et al., 2016; Philippi et al., 2015). Nevertheless, there remains a significant gap in the literature regarding resting-state FC across the four major brain networks in individuals with StD, with a notable paucity of large-sample clinical studies on StD.

Depression is now conceptualized as a spectrum that includes subthreshold symptoms to severe manifestations (Rodriguez et al., 2012). The relationship between cognitive dysfunction and depression is a critical area of investigation in both clinical depression and StD. Evidence from two cross-sectional studies suggests that StD is predominantly characterized by cognitive and mood symptoms, rather than somatic or vegetative symptoms (Howland et al., 2008; Rapaport et al., 2002). Cognitive-affective symptoms, such as difficulties in concentration, are particularly salient and may play a significant role in the development of StD (Liao et al., 2022). Individuals with StD demonstrate more significant deficits across various cognitive domains, including reduced performance in letter fluency (Dotson, Resnick, & Zonderman, 2008), cognitive control (Dotson et al., 2020), processing speed (Forbes et al., 2024), and reward processing (Mori et al., 2016). Previous studies often utilized heterogeneous cognitive measures, resulting in incomplete and unsystematic assessments of the cognitive functioning associated with StD. The MATRICS Consensus Cognitive Battery (MCCB) has proven effective in providing valuable insights into the cognitive characteristics of individuals with mood disorders (Liang et al., 2020; Nuechterlein et al., 2008). Documented impairments encompass attention, working memory, processing speed, verbal and visual learning, social cognition, and reasoning/problem-solving abilities (Lai et al., 2022). A recent meta-analysis suggests that first-degree relatives of patients with MDD are at an increased risk for cognitive impairment, identifying cognitive dysfunction as a significant endophenotype for MDD (MacKenzie, Uher, & Pavlova, 2019). Persistent subsyndromal depressive symptoms significantly contribute to ongoing cognitive impairment (Preiss et al., 2009; Weiland-Fiedler et al., 2004). To date, no studies have investigated cognitive impairment in individuals with StD using MCCB, and no large-scale research has been published on this topic.

This study utilized fMRI to explore FC in individuals with StD and MDD, with a focus on seeds from the DMN, ECN, SN, and AN. Building on prior research that has identified abnormal FC patterns in individuals with depression, we hypothesized that individuals with StD might exhibit atypical FC patterns across various brain functional networks, akin to those observed in MDD. Additionally, StD may present unique alterations in FC within these networks, potentially providing insights into the pathophysiology of StD. We also examined the hypothesis that network properties are associated with cognitive impairments in individuals with StD.

## Materials and methods

### Participants

A total of 153 individuals with StD (55 males, 98 females; mean age, 22.11 ± 2.88 years) were recruited from Jinan University, Guangzhou, China. StD is characterized by at least one key symptom of depression without meeting the full criteria of the Diagnostic and Statistical Manual of Mental Disorders (Fifth Edition) (DSM-5) for the diagnosis of depression or other psychiatric disorders. Ages between 18 and 45 years were required for participants to minimize age-related vascular lesions and degenerative brain lesions. In order to assess clinical state, the Center for Epidemiologic Studies Depression Scale (CESD), Young Mania Rating Scale (YMRS), and 24-item Hamilton Depression Rating Scale (24-item HDRS) were used. All enrolled StD participants were required to have an HDRS score between 8 and 20 points (Demyttenaere & De Fruyt, 2003), a CESD score ≥ 16 points (Buntrock et al., 2016; Jiang et al., 2019), and a YMRS score of <7 points.

A total of 188 outpatients or inpatients (69 males, 119 females; mean age, 22.71 ± 4.21 years) diagnosed with MDD were recruited from the psychiatry department at the First Affiliated Hospital of Jinan University, Guangzhou, China. This study used the Structured Clinical Interview for DSM-IV Patient Edition (SCID-P) to diagnose MDD according to the criteria of the DSM-5. No medication was given to any of the enrolled patients, and all had an HDRS score of ≥21 and a YMRS score of <7 points.

A total of 110 HCs (48 males and 62 females; mean age, 22.38 ± 3.20 years) were recruited from local advertisements and were matched for age and gender. The Structured Clinical Interview for DSM-5 Nonpatient Edition (SCID-NP) was used to meticulously screen all participants. HCs were required to have a CESD score of < 16 points, an HDRS score of <8 points, and a YMRS score of <7 points.

Exclusion criteria for both StD and HCs included any psychiatric illness, family history of psychiatric illness, significant neurological and medical conditions, pregnancy, and MRI contraindications. Exclusion criteria for MDD included: (i) current psychiatric illness, (ii) history of organic or neurological brain illness, (iii) substance or alcohol abuse or dependence, (iv) history of electroconvulsive therapy, (v) physical illness identified through laboratory or clinical examinations or personal history, and (vi) pregnancy or MRI contraindications.

Two experienced clinical psychiatrists diagnosed all participants. All participants were of Chinese Han ethnicity and right-handed. They received a detailed verbal and written explanation of the study prior to signing informed consent forms. The study received approval from the Ethics Committee of the First Affiliated Hospital of Jinan University, Guangzhou, China, in compliance with the Declaration of Helsinki.

#### Procedure

Experienced physicians conducted all cognitive and clinical assessments. Clinical assessments were conducted within the 2 days preceding the cognitive evaluation and MRI scan.

#### Clinical assessment

All participants underwent the following evaluations. The HDRS was used to measure depression severity, while the YMRS was used to measure manic symptoms. The StD group and HCs also used the CESD to assess depressive symptoms.

#### Cognitive assessment

Chinese versions of the MCCB, consisting of nine subtests, were used in neuropsychiatric assessments. The MCCB identifies seven cognitive domains: speed of processing, attention/vigilance, working memory, verbal learning, visual learning, reasoning/problem-solving, and social cognition. Participants underwent testing in a consistent sequence. Cognitive assessments were conducted in a quiet environment and lasted approximately 60 minutes. Standardized scores were calculated based on age, gender, and years of education, integrating the original test scores into the MCCB scoring system.

### MRI data acquisition and preprocessing

MRI data were obtained using a 3.0 T MRI scanner (GE Healthcare, Discovery MR750) equipped with an eight-channel phased array head coil. Cushions were placed inside the coil to minimize head movement while scanning in the supine position. During the scanning procedure, participants were instructed to keep their eyes closed and avoid falling asleep. A total of 153 StD individuals, 188 MDD patients, and 110 HCs were included. Participants with poor-quality scans or incomplete imaging data were excluded to ensure accurate clinical–imaging correlations.

A time repetition (TR) of 2000 ms and a time echo (TE) of 25 ms were used for the acquisition of rs-fMRI data with a gradient-echo, echo-planar imaging (EPI) sequence. The parameters included a 90°flip angle, a 64 × 64 matrix, a 240 × 240 mm^2^ field of view (FOV), a voxel size of 3.75 × 3.75 × 3 mm^3^, a slice thickness/gap of 3.0/1.0 mm, and 210 volumes acquired in 7 minutes from 35 axial slices covering the whole brain. To obtain structural data, a three-dimensional (3D) brain volume imaging (BRAVO) sequence was acquired with the following parameters: TR/TE 8.2/3.2 ms, flip angle 12°, matrix 256 × 256, FOV 240 × 240 mm^2^; slice thickness/gap 1.0/0 mm, bandwidth 31.25 Hz, NEX 1, and an acquisition time of 3 min 45 s. A routine T2-weighted image was acquired to exclude organic brain abnormalities, with all image acquisitions conducted by two experienced neuroradiologists.

### Data preprocessing

Based on Statistical Parametric Mapping (SPM12), the Data Processing Assistant for Resting-State fMRI (DPABI_V3.0) was used to preprocess the rs-fMRI data. A consistent longitudinal magnetization was maintained by excluding the initial 10 images from the rs-fMRI dataset. Following slice-timing correction, the remaining 200 images were realigned to the initial image to correct for head motion during TR. With this realignment adjustment, a record of head movement was obtained during the rs-fMRI scan. Each subject’s displacement was required to remain within 2 mm in any plane, 2° in angular motion, or a mean frame-wise displacement (FD) of 0.2 mm (Jenkinson, Bannister, Brady, & Smith, 2002). Using a segmentation toolbox, the T1 structural images were classified as gray matter, white matter, and cerebrospinal fluid. A template specific to the study was generated using the DARTEL toolbox to ensure precise normalization. The structural images were used to co-register and transform the resting-state functional images into standard Montreal Neurological Institute (MNI) space. The voxel size was adjusted to a resolution of 3 × 3 × 3 mm^3^ by reslicing. The data were detrended and then filtered between 0.01 Hz and 0.1 Hz using a band-pass filter. The time course of each voxel was refined by eliminating extraneous variables and their temporal derivatives, such as the global brain mean, white matter, cerebrospinal fluid signals, and the Friston-24 head motion parameters (consisting of 6 head motion parameters from the previous time point and the corresponding 12 squared terms) (Friston et al., 1996; Power et al., 2014). A 6-mm full-width at half-maximum (FWHM) Gaussian kernel was used for smoothing.

### Resting-state functional connectivity

Based on prior research (Dunlop et al., 2023), we selected eight spherical regions of interest (ROIs) with a 5-mm radius as seeds for the DMN, ECN, SN, and AN. These ROIs include the PCC [MNI coordinates: ±7, −43, +33], dorsolateral prefrontal cortex (DLPFC) [MNI coordinates: ±36, +27, +29], anterior insula (AI) [MNI coordinates: ±36, +18, +4], and subcallosal cingulate cortex (SCC) [MNI coordinates: ±6, +24, −11] (Supplementary Figure S1). Voxel-wise correlations were performed between the seed mean time course and the whole-brain mean time course. Pearson’s correlation coefficients were calculated between the average time series of the bilateral seed regions and the voxel time series in each brain region to create individual rs-FC maps. Fisher’s transformation was subsequently applied to transform the subject-level correlation maps into z-value maps, enhancing normality. In the end, we acquired a total of eight z-score maps that depict the inherent FC of the ROIs across all subjects. In all FC maps, a Gaussian kernel of 6 mm FWHM was used for smoothing.

### Statistical analysis

Data analysis was conducted using SPSS 24.0 software (SPSS, Chicago, IL, USA). The Kolmogorov–Smirnov goodness-of-fit test was used to assess the normality of all variables. One-way ANOVA was used to analyze normally distributed clinical scales (excluding CESD), demographic data (excluding gender), and cognitive variables, while the Kruskal–Wallis test was applied to skewed variables. We then used a Bonferroni post hoc test to determine whether the results were significant. An independent two-sample *t*-test compared CESD differences between the StD group and HCs. A chi-squared test compared gender distribution among the three groups. Two-tailed tests with Bonferroni correction for *p*-values were employed.

Effect sizes were represented as *Z*-scores with a mean of zero and a standard deviation of one. *Z*-scores for the StD, MDD groups, and HCs were calculated using the equation: (raw score - mean _control group_) / SD _control group_.

A two-sample *t*-test was conducted on *z*-score maps for each seed to assess the statistical significance of within-group FC in StD, MDD, and HCs. An uncorrected *p*-value below 0.05 was deemed statistically significant. We conducted a one-way ANOVA to evaluate significant differences in whole-brain FC across regions among the three groups, using the union mask from the two-sample *t*-test. When comparing whole-brain FC among groups, demographic data such as age, gender, education level, and mean frame-wise displacement (FD) were included as nuisance covariates. We applied Gaussian random field (GRF) theory to correct for multiple comparisons (voxel-wise *p* < 0.005; cluster significance *p* < 0.0125, GRF corrected). Brain regions showing significant differences in one-way ANOVA (LSD corrected, *p* < 0.05) were designated as ROIs for subsequent post-hoc pairwise group analysis.

Partial correlation analysis examined the relationships between abnormal FC values, cognitive function, and HDRS in StD individuals and MDD patients. Age, gender, and education level were controlled. Bonferroni correction was again applied to these correlational analyses. Moreover, StD individuals and MDD patients were analyzed together for correlations among abnormal FC values, cognitive function, and HDRS scores using partial correlation analysis. Age, gender, educational level, 24-item HDRS, and CESD scores were controlled. Bonferroni correction was again applied to these correlational analyses. The associations between FC values, cognitive function, and HDRS score were explored using PROCESS v3.4.1 in SPSS through a mediation analysis. The confidence intervals in the output have a 95% confidence level, and 5000 bootstrap samples were used.

### Support vector machine (SVM) analysis

LIBSVM (https://www.csie.ntu.edu.tw/~cjlin/libsvm/) was used to determine whether distinct or combined features of significantly different FC and cognitive function could differentiate subjects with StD from HCs and patients with MDD from HCs. A Gaussian radial basis function (GRBF) SVM model optimally separates distinct feature classes using a hyperplane. A grid search was conducted to identify the optimal penalty coefficient (*c*) and gamma (*g*). Furthermore, the ‘k-fold’ (*k* = 10) cross-validation technique and a permutation test (5,000 times) were employed to achieve optimal accuracy, sensitivity, and specificity (*p* < 0.05). The area under the receiver operating characteristic curve (AUC) was used to assess each model’s classification performance. The separate or fused features were divided into k blocks, and the software automatically randomly assigned all subjects to each block.

## Results

### Demographic and clinical information

Table 1 provides a summary of the demographics and clinical characteristics of the study participants. The MDD group had significantly fewer years of education compared to the StD group (*p* = 0.002) and HCs (*p* < 0.001). Both the StD and MDD groups reported significantly higher 24-item HDRS scores compared to HCs (both *p* < 0.001), and the MDD group also scored higher than the StD group (*p* < 0.001). The StD group had a higher CESD score than the HCs (*p* < 0.001). In addition, the YMRS score for MDD was significantly higher than that for both StD and HCs (both *p* < 0.001).

**Table 1.** Demographics and clinical data of all participants [mean (SD)]

	StD group	MDD group	HCs	*F*, *X*^*2*^, *Z*, *t*	*p-value*	*p-value*^*e*^
Number of subjects	153	188	110	–	–	
Age (year)	22.11 (2.88)	22.71 (4.21)	22.38 (3.20)	1.237	0.291^a^	
Age range (year)	18–45	18–45	18–45	–	–	
Gender (male/female)	55/98	69/119	48/62	1.885	0.390^b^	
Education (year)	15.60 (2.47)	14.63 (2.07)	15.76 (2.58)	19.151	< 0.001^c*^	MDD < StD, HCs^e^
24-item HDRS score	13.87 (4.00)	25.92 (4.85)	2.38 (1.98)	376.311	<0.001^c*^	MDD > StD > HCs^e^
CESD score	26.47 (7.12)	–	5.53 (4.22)	29.58	<0.001^d*^	
YMRS score	0.74 (0.48)	2.57 (2.01)	0.45 (0.88)	31.884	<0.001^c*^	MDD < StD, HCs^e^

*Note*: StD, subthreshold depression; MDD, major depressive disorder; HCs, healthy controls; HDRS, Hamilton Depression Rating Scale; CESD, center for epidemiological survey depression scale; YMRS, Young Mania Rating Scale; ^a^, One-Way ANOVA analyses; ^b^, X^2^ test; ^c^, Kruskal–Wallis test; ^d^, Independent-sample *t*-test; ^e^, Bonferroni post hoc test; *, *p*<0.05 significant.

### Cognitive function analysis

**Figure 1.**
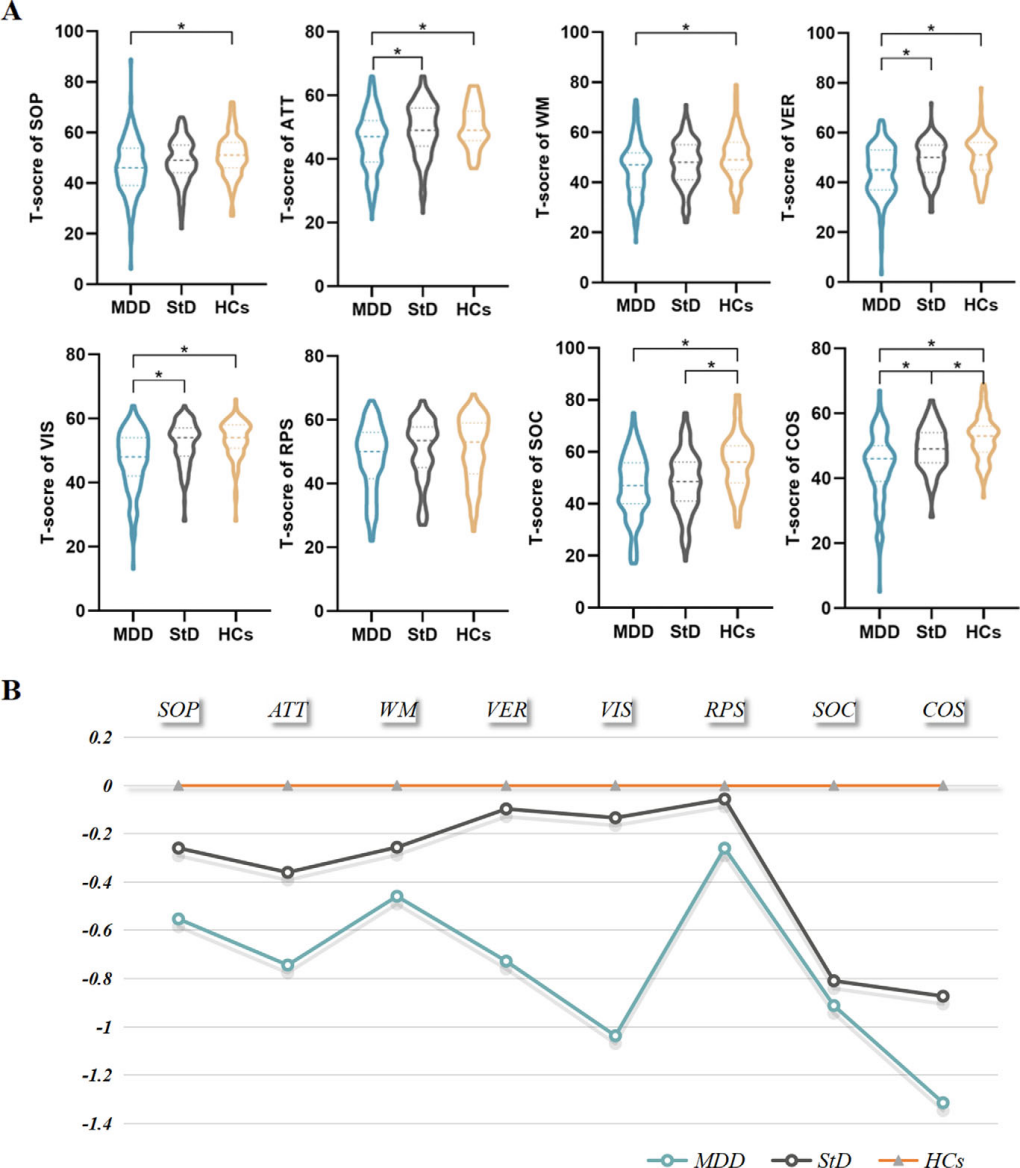
MCCB performance in StD, MDD, and HCs. (a) The violin plots of comparison of MCCB among the three groups. (b)The x-axis indicates the seven MCCB domains and the composite score. The y-axis depicts a Z-score with a mean of zero and an SD of 1. StD, subthreshold depression; MDD, major depressive disorder; HCs, healthy controls; SOP, speed of processing; ATT, attention/vigilance; WM, working memory; VER, verbal learning; VIS, visual learning; PRS, reasoning/problem-solving; SOC, social cognition; COS, composite.

The analysis included 124 StD individuals, 140 MDD patients, and 90 HCs, as 29 StD individuals, 48 MDD patients, and 20 HCs failed to complete the cognitive assessment. StD, MDD, and HCs were successfully matched for age, gender, and years of education. Participants’ demographic and clinical data are available in Supplementary Table S1. In Table 2 and Figure 1, we compare cognitive function indices among the three groups. Compared to HCs, the StD group exhibited significant impairments in social cognition and composite scores (both *p* < 0.05), and the MDD group exhibited significant deficits in processing speed, attention/vigilance, working memory, verbal learning, visual learning, social cognition, and composite scores (all *p* < 0.05) (Figure 1a). The MDD group exhibited significant impairments in attention/vigilance, verbal learning, visual learning, and composite scores compared to the StD group (all *p* < 0.05). Compared to the MCCB normative data of HCs, individuals with StD showed effect sizes ranging from −0.81 to −0.06 SDs below average across all seven MCCB domains, with a composite score of −0.87 SDs below average. The MDD group exhibited below-average effect sizes across all seven MCCB domains, ranging from −1.04 to −0.26 SDs, with the MCCB composite score being −1.32 SDs lower (Figure 1b).

**Table 2.** Comparisons of cognitive function indices among StD, MDD, and HCs group [mean (SD)]

	StD group (*n* = 124)	MDD group (*n* = 140)	HCs (*n* = 90)	*F*	*p-value*	*p-value*^*e*^
Speed of processing	48.60 (8.42)	46.14 (11.01)	50.77 (8.38)	13.176	0.001^c*^	MDD < HCs^e^
Attention/vigilance	49.24 (8.30)	45.47 (9.06)	50.13 (6.70)	18.907	< 0.001^c*^	MDD < StD, HCs^e^
Working memory	47.52 (9.14)	45.54 (10.77)	50.02 (9.75)	5.554	0.004^a*^	MDD < HCs^e^
Verbal learning	49.29 (7.14)	44.27 (10.28)	50.11 (8.45)	24.617	< 0.001^c*^	MDD < StD, HCs^e^
Visual learning	53.01 (5.76)	46.99 (9.36)	53.40 (6.13)	43.598	< 0.001^c*^	MDD < StD, HCs^e^
Reasoning/problem-solving	50.61 (9.85)	48.14 (10.60)	50.77 (10.12)	2.600	0.076^a^	
Social cognition	48.06 (11.88)	46.55 (12.19)	56.05 (10.91)	19.082	< 0.001^a*^	MDD, StD < HCs^e^
Composite	49.26 (6.19)	43.78 (10.43)	52.44 (6.82)	47.409	< 0.001^c*^	MDD < StD < HCs^e^

*Note*: StD, subthreshold depression; MDD, major depressive disorder; HCs, healthy controls; ^a^, One-Way ANOVA analyses; ^c^, Kruskal–Wallis test; ^e^, Bonferroni post hoc test; *, *p* < 0.05 significant.

### FC analysis

**Figure 2.**
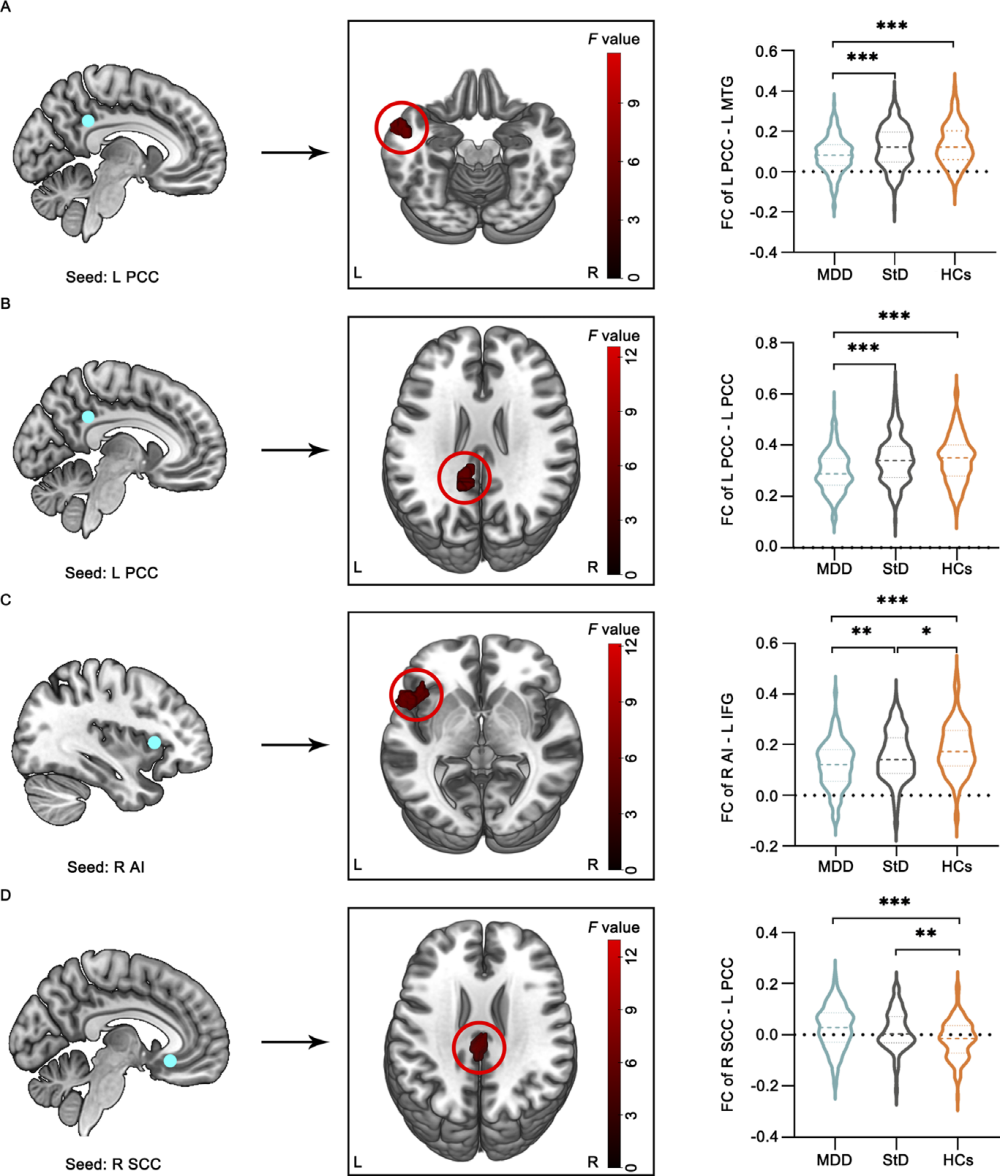
The significant FC differences among the three groups for the seeds (voxel *p* < 0.005, cluster *p* < 0.0125, GRF corrected). The color bar indicates the *F* values from one-way ANOVA analyses. *, *p*<0.05 significant; **, *p*<0.01 significant; ***, *p*<0.001 significant; StD, subthreshold depression; MDD, major depressive disorder; HCs, healthy controls; FC, functional connectivity; GRF, Gaussian random field; BA, Brodmann Area; MNI, Montreal neurological coordinate; PCC, posterior cingulate cortex; MTG, middle temporal gyrus; AI, anterior insula; IFG, inferior frontal gyrus; SCC, subcallosal cingulate cortex; L/R, left/right hemisphere.

A two-sample *t*-test identified within-group FC patterns in the three groups (Supplementary Figure S9, *p* < 0.05, uncorrected, for visual inspection) for each brain regional seed. Table 3 and Figure 2 present the FC value comparisons of the three groups for each seed (voxel *p*-value <0.005; cluster significance: *p* < 0.0125, GRF corrected). In order to assess the differences among the three groups, we conducted a post hoc analysis (*p* < 0.005, LSD correction). Both the StD and MDD groups showed reduced FC between the right AI and the left inferior frontal gyrus (IFG), and increased FC between the right SCC and the left PCC (both *p* < 0.05) compared to HCs. Additionally, the MDD group exhibited significantly decreased FC between the left PCC and the left MTG (*p* < 0.001), and between the left PCC and the left PCC (*p* < 0.001), compared to HCs. The StD group showed significantly higher FC between the left PCC and the left MTG (*p* < 0.001), the left PCC and the left PCC (*p* < 0.001), and the right AI and the left IFG (*p* = 0.002), compared to the MDD group.

**Table 3.** The significant FC difference among StD, MDD, and HCs group (voxel p < 0.005, cluster p < 0.0125, GRF corrected)

seeds	One-way ANOVA (voxel *p* < 0.005, cluster *p* < 0.0125, GRF corrected)						*F*		
	Significant regions	BA	voxels	MNI				Post hoc analysis (LSD correction, *p* < 0.05)	
				x	y	z		Comparisons	*p*
L PCC	L MTG	21	57	−51	−6	−21	11.751	MDD < HCs MDD < StD	< 0.001 < 0.001
L PCC	L PCC	23	118	−12	−39	30	12.871	MDD < HCs MDD < StD	< 0.001 < 0.001
R AI	L IFG	38	90	−47	17	−6	9.197	MDD < HCs MDD < StD StD < HCs	< 0.001 0.002 0.025
R SCC	L PCC	23	49	−2	−26	26	9.182	MDD > HCs StD > HCs	< 0.001 0.002

*Note*: StD, subthreshold depression; MDD, major depressive disorder; HCs, healthy controls; FC, functional connectivity; GRF, Gaussian random field; BA, Brodmann Area; MNI, Montreal Neurological coordinate; PCC, posterior cingulate cortex; MTG, middle temporal gyrus; AI, anterior insula; IFG, inferior frontal gyrus; SCC, subcallosal cingulate cortex; L/R, left/right hemisphere.

### Correlation and mediation analysis

**Figure 3.**
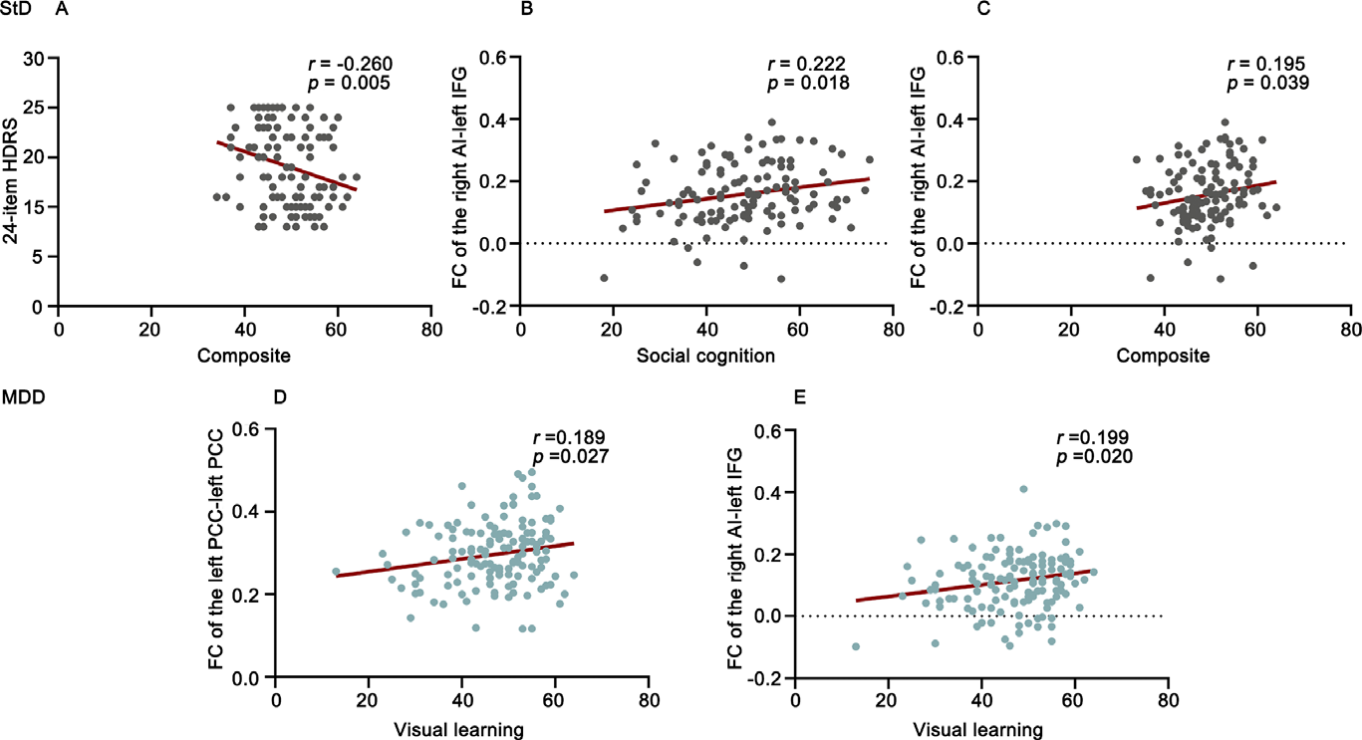
The correlations among significant FC values, cognitive function, and HDRS in StD individuals and MDD patients. StD, subthreshold depression; MDD, major depressive disorder; HDRS, Hamilton Depression Rating Scale; FC, functional connectivity; PCC, posterior cingulate cortex; AI, anterior insula; IFG, inferior frontal gyrus.

Figure 3 presents the correlations between abnormal FC values, cognitive function, and HDRS in individuals with StD and patients with MDD. In StD, the 24-item HDRS score showed a negative correlation with the composite score (*r* = −0.296, *p* = 0.005). Additionally, FC in the right AI–left IFG was positively correlated with social cognition (*r* = 0.222, *p* = 0.018) and the composite (*r* = 0.195, *p* = 0.039). In MDD, visual learning was positively correlated with FC in the left PCC–left PCC (*r* = 0.189, *p* = 0.027) and FC in the right AI–left IFG (*r* = 0.199, *p* = 0.020).

**Figure 4.**
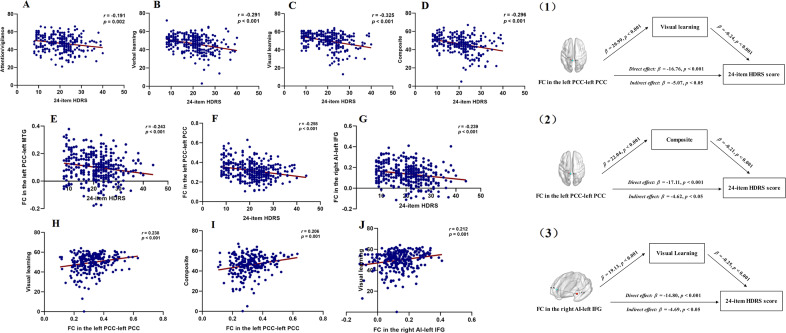
The results of correlation analysis and mediation analysis for analyzing StD individuals and MDD patients together. A–J shows the correlations among abnormal FC values, cognitive function, and HDRS in MDD patients and StD individuals, using partial correlation analysis. (1)–(3) shows the associations between FC values, cognitive function, and HDRS score in MDD patients and StD individuals, using mediation analysis. HDRS, Hamilton Depression Rating Scale; FC, functional connectivity; PCC, posterior cingulate cortex; AI, anterior insula; IFG, inferior frontal gyrus.

Figure 4 shows the correlations among abnormal FC values, cognitive function, and HDRS scores in MDD patients and StD individuals. The 24-item HDRS score showed negative correlations with the attention/vigilance (*r* = −0.191, *p* = 0.002), verbal learning (*r* = −0.291, *p* < 0.001), visual learning (*r* = −0.325, *p* < 0.001), composite scores (*r* = −0.296, *p* < 0.001), FC in the left PCC–left MTG (*r* = −0.243, *p* < 0.001), FC in the left PCC–left PCC (*r* = −0.258, *p* < 0.001), and FC in the right AI–left IFG (*r* = −0.239, *p* < 0.001). In addition, FC in the left PCC–left PCC was positively correlated with visual learning (*r* = 0.238, *p* < 0.001) and composite scores (*r* = 0.206, *p* = 0.001), and FC in the right AI–left IFG was positively correlated with visual learning (*r* = 0.212, *p* = 0.001).

In mediation analysis, we constructed hypothetical models of relationships among abnormal FC values, cognitive function, and depressive symptom severity in MDD patients and StD individuals. The direct (*β* = −16.76, *p* < 0.001) and indirect effects (*β* = −5.07, *p* < 0.05) of FC in the left PCC–left PCC on the 24-item HDRS score were statistically significant, indicating that visual learning partially mediated the relationship between FC in the left PCC–left PCC and the 24-item HDRS score. The direct (*β* = −17.11, *p* < 0.001) and indirect effects (*β* = −4.62, *p* < 0.05) of FC in the left PCC–left PCC on the 24-item HDRS score were also statistically significant, indicating that the composite score partially mediated the relationship between FC in the left PCC–left PCC and the 24-item HDRS score. Additionally, the direct (*β* = −14.80, *p* < 0.001) and indirect effects (*β* = −4.69, *p* < 0.05) of FC in the right AI–left IFG on the 24-item HDRS score were statistically significant, indicating that the composite score partially mediated the relationship between FC in the right AI–left IFG and the 24-item HDRS score.

### SVM analysis

**Figure 5.**
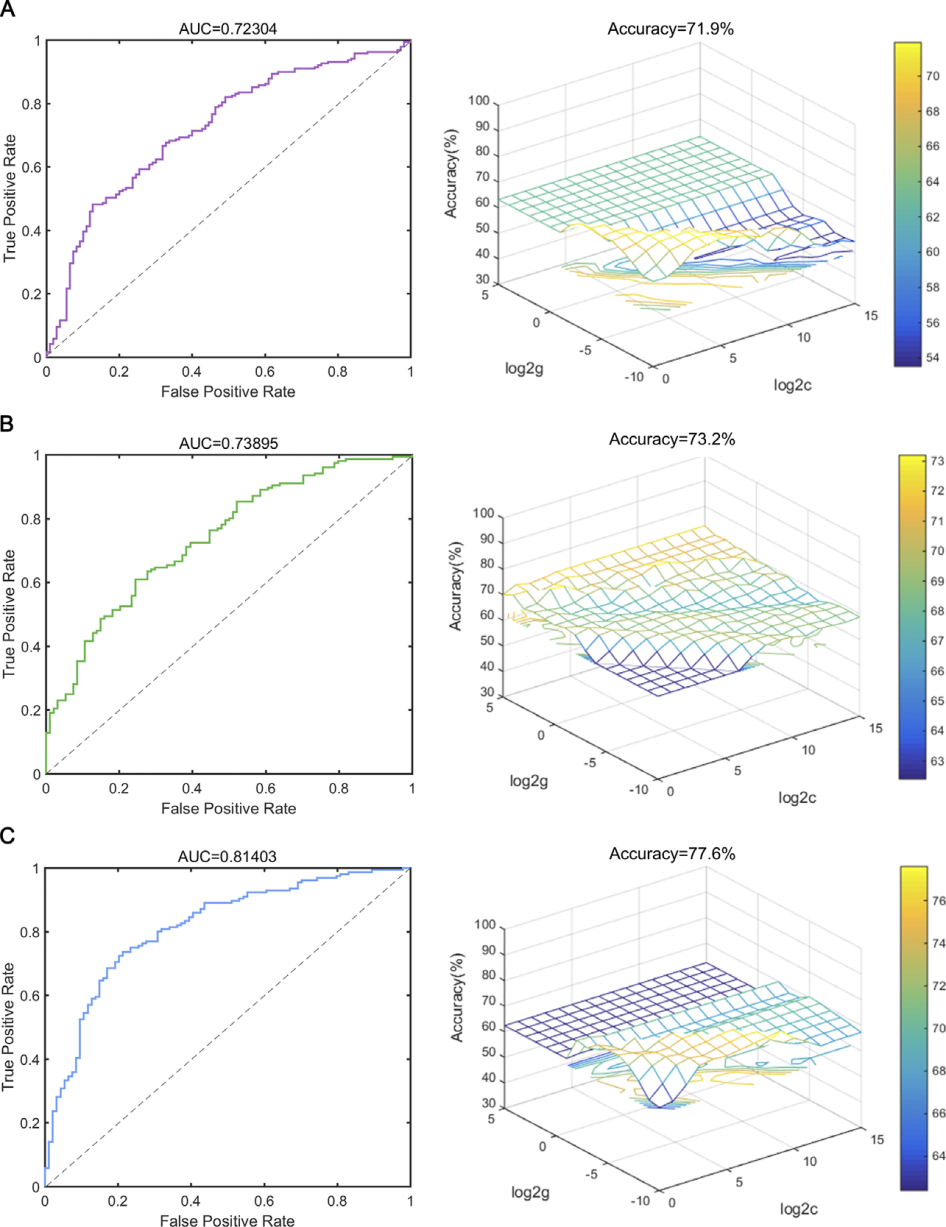
SVM classification performance in distinguishing MDD from HCs and StD from HCs. (A) The ROC curves and 3D view of the classified accuracy of fusion features of the FC in distinguishing MDD from HCs with the best parameters (best *C* = 16, *g* = 0.002). (B) The ROC curves and 3D view of the classified accuracy of fusion features of thecognitivefunction in distinguishing MDD from HCs with the best parameters (best *C* = 4, *g* = 8). (C) The ROC curves and 3D view of the classified accuracy of fusion features of FC and cognitive function in distinguishing MDD from HCs with the best parameters (best *C* = 512, *g* = 0.001). (D) The ROC curves and 3D view of the classified accuracy of fusion features of the FC in distinguishing StD from HCs with the best parameters (best *C* = 16, *g* = 0.004). (E) The ROC curves and 3D view of the classified accuracy of fusion features of the cognitive function in distinguishing StD from HCs with the best parameters (best *C* = 64, *g* = 32). (F) The ROC curves and 3D view of the classified accuracy of fusion features of FC and cognitive function in distinguishing StD from HCs with the best parameters (best *C* = 128, *g* = 0.002). SVM, support vector machine; MDD, major depressive disorder; StD, subthreshold depression; HCs, healthy controls; FC, functional connectivity; AUC, area under the curve.

The results of SVM classification based on distinct or fused features of significantly different FC and cognitive function for differentiating MDD from HCs and StD from HCs are displayed in the Figure 5 and Table 4. The fused features of significantly different FC and cognitive function showed the best performance in classifying MDD from HCs (with an AUC of 0.814, an accuracy of 77.60%, a specificity of 60.64%, and a sensitivity of 83.33%). Meanwhile, the results of SVM classification for differentiating StD from HCs (with an AUC of 0.677, an accuracy of 66.4%, a specificity of 56.38%, and a sensitivity of 76.19%) are also displayed.

**Table 4.** Support vector machine (SVM) classification performance of distinct and fusion features in distinguish MDD from HCs and StD from HCs

	Accuracy (%)	Sensitivity (%)	Specificity (%)	AUC
MDD from HCs				
FC	71.9	83.07	48.18	0.723
Cognitive function	73.2	79.49	50.00	0.739
FC+ Cognitive function	77.6	83.33	60.64	0.814*
StD from HCs				
FC	61.6	73.86	30.00	0.545
Cognitive function	67.3	75.40	47.87	0.675
FC+ Cognitive function	66.4	76.19	56.38	0.677

*Note*: MDD, major depressive disorder; StD, subthreshold depression; HCs, healthy controls; AUC, area under the curve; *, achieved the best classification performance.

## Discussion

This study investigated the variations in brain network connectivity and cognitive function among individuals with StD, MDD, and HCs. The results revealed that individuals with StD exhibited deficits solely in social cognition, whereas those with MDD demonstrated impairments across multiple cognitive domains relative to HCs. Both the StD and MDD groups displayed similarly altered connectivity patterns between the AI and the IFG, as well as between the SCC and the PCC, compared to HCs. Notably, patients with MDD exhibited particularly decreased connectivity between the PCC and the MTG, as well as within the left PCC, while no abnormal FC of the DMN was observed in individuals with StD. Correlation analyses revealed a positive association between the FC of the right AI and the left IFG with social cognition and composite scores in individuals with StD. In MDD patients, visual learning was positively correlated with FC within the left PCC and between the right AI and the left IFG.

Our findings indicate that individuals with StD and patients with MDD exhibit reduced FC between the right AI and the left IFG compared to HCs, suggesting similar aberrant FC of the SN in both StD and MDD. The AI is a component of the SN, and the IFG is a critical region within the prefrontal lobe, often referred to as a ‘multifunctional’ area essential for emotional, behavioral, and cognitive regulation (Gabrieli, Poldrack, & Desmond, 1998). Alterations in this network may signal the need for enhanced processing and trigger appropriate cognitive control mechanisms (Williams, 2016). Previous fMRI studies have demonstrated a significant reduction in the degree of centrality of the orbital portion of the left IFG in individuals with StD (Huang et al., 2024). The IFG and AI are involved in dynamic attentional and working memory processes (Tops & Boksem, 2011) and are frequently associated with increased activity in response to anxiety and distress (Etkin & Wager, 2007). Antidepressant treatment has been shown to result in a shift in activation from the IFG/AI to the DLPFC (Wu et al., 2008). Our findings highlight the critical role of abnormal SN activity in the pathophysiology of StD and MDD. Additionally, the StD group exhibited significantly greater FC between the right AI and the left IFG compared to the MDD group. This finding suggests that alterations in SN connectivity may be involved in the earliest functional abnormalities associated with StD. Prior research indicates that the connectivity strength in individuals with StD is intermediate between that observed in MDD patients and HCs, implying that StD could represent a transitional phase between a healthy state and MDD (T. Zhang et al., 2020). The findings from the present study corroborate this hypothesis.

Additionally, this study identified increased FC between the right SCC and the left PCC in both the StD and MDD groups relative to HCs. Given its anatomical connections with frontal, limbic, and paralimbic structures (Johansen-Berg et al., 2008), the SCC, a component of the AN, is posited to be at the intersection of cognitive and affective processing. Dysfunction in this region has been linked to impaired emotion regulation (Mayberg, 1997; Seminowicz et al., 2004). The overactivation of the SCC has been shown to induce anticipatory and motivational anhedonia (Alexander et al., 2019), a prominent symptom of depression, particularly among patients with treatment-resistant depression (Kelly, Freeman, & Schumacher, 2022). Patients with MDD have exhibited increased cerebral blood flow in the SCC during depressive relapse (Hasler et al., 2008; Neumeister et al., 2004), which decreases upon remission (Holthoff et al., 2004). Previous study has consistently observed that SCC-PCC connectivity is elevated in MDD patients compared to HCs during resting states (Ho et al., 2015), suggesting a persistent elevation of DMN connections associated with depression. Animal studies have indicated a positive correlation between SCC activity and elevated plasma cortisol levels (Banai Tizkar, McIver, Wood, & Roberts, 2024; Jahn et al., 2010). This may be attributed to increased expression of mineralocorticoid (MR) and glucocorticoid (GR) receptors (Patel et al., 2000), rendering the SCC highly sensitive to stress-related effects, including circulating cortisol. Furthermore, the SCC may influence cortisol release through its projections to the hypothalamus (Joyce & Barbas, 2018). Consequently, the observed increase in connectivity between the SCC and the PCC suggests that individuals with StD may exhibit FC patterns between the AN and the DMN similar to those observed in individuals diagnosed with MDD. This dysfunction may play a critical role in the pathogenesis and progression of the disorder.

Furthermore, in comparison to HCs, patients with MDD demonstrated notably reduced connectivity between the PCC and the MTG, as well as within the left PCC, while the DMN FC did not differ significantly between individuals with StD and HCs. The StD group exhibited significantly higher FC between the left PCC and the left MTG, and within the left PCC itself, compared to the MDD group. The PCC and MTG are essential nodes within the DMN (Smallwood et al., 2021). These findings suggest that individuals with StD display distinct connectivity patterns within the DMN compared to those with MDD. The DMN has garnered substantial interest in clinical neuroscience, particularly in relation to depression, largely due to its association with self-referential processes, which offer an intuitive framework for understanding the neural mechanisms underlying rumination in MDD (Fox et al., 2005). Previous neuroimaging research has demonstrated the pivotal role of the PCC in the retrieval of autobiographical and self-relevant information (Cavanna, 2007; Spreng, Mar, & Kim, 2009). Furthermore, increased PCC activation has been positively associated with elevated levels of depression and hopelessness (Grimm et al., 2009). The MTG has also been implicated in behavioral disturbances associated with MDD, particularly in relation to rumination (Nejad, Fossati, & Lemogne, 2013; Sheline, Price, Yan, & Mintun, 2010) and emotional processing (Cao et al., 2014). A study conducted by the REST-meta-MDD Consortium, which analyzed a substantial fMRI dataset of MDD patients, identified that reductions in DMN FC occur exclusively in patients with recurrent MDD, as opposed to those experiencing a first-episode drug-naïve MDD (Yan et al., 2019). Additionally, DMN FC was found to be positively correlated with symptom severity only in recurrent MDD cases (Yan et al., 2019). These findings imply that StD represents an early developmental stage of depression that is insufficient to induce alterations in DMN connectivity, and that aberrant DMN connectivity may be associated with the pathogenesis and recurrence of major depression.

Additionally, individuals with StD were subjected to a comprehensive neurocognitive test designed to evaluate cognitive performance across various domains, a research area previously unexplored within this population. In alignment with prior studies (Lai et al., 2022; Y. Zhang et al., 2024), individuals with MDD demonstrated reduced cognitive performance across multiple domains, including processing speed, working memory, attention, verbal and visual learning, and social cognition, when compared to HCs. In contrast, individuals with StD exhibited significant impairments solely in the domain of social cognition relative to HCs. These findings suggest that social cognition is the primary and exclusive cognitive domain affected in individuals with StD. Social cognition involves the cognitive processes that allow individuals to understand their own and others’ thoughts, feelings, and intentions (Mitchell & Phillips, 2015), which are essential for effective functioning in both professional and community contexts (Fett et al., 2011). Previous research suggests that alterations in social cognition contribute to the social difficulties associated with depression (Ladegaard, Larsen, Videbech, & Lysaker, 2014; Weightman, Air, & Baune, 2014). Deficits in fundamental social cognition can hinder the development and functioning of more complex skills (Porter-Vignola et al., 2022). Research suggests that the severity of depressive symptoms influences the relationship between social cognition and social functioning (Lahera et al., 2015). Moreover, subthreshold depression has the potential to disrupt normal social cognition, particularly affecting first-order cognitive theory-of-mind functioning (Navarra-Ventura et al., 2021). Our findings indicate that patients with MDD exhibited multidimensional cognitive impairments, whereas deficits in social cognition were the initial manifestation in individuals with StD.

Correlation analyses conducted in individuals with StD revealed a significant positive correlation between social cognition and FC in the right AI and the left IFG. The PCC is integral to cognitive functions such as spatial attention, episodic memory, and self-evaluation (Greicius, Krasnow, Reiss, & Menon, 2003). The AI facilitates dynamic interactions between large-scale brain networks responsible for external attention and self-cognition (Singer, Critchley, & Preuschoff, 2009). In healthy individuals, activation of the SN is frequently observed during various cognitive tasks (Menon & Uddin, 2010). As previously discussed, social cognition may decline before the onset of depression, a condition closely linked to abnormal connectivity between the SN and the IFG. Our study identifies a positive correlation between visual learning in patients with MDD and FC between the right AI and left IFG, as well as between the left PCC and left PCC. Visual learning deficits in individuals with mental illness reflect the difficulties associated with updating and adapting to multiple sensory inputs within a limited timeframe (Andrade et al., 2016), thereby contributing to their social cognitive impairments (Lu et al., 2021). Our findings indicate that patients with MDD exhibit decreased FC between the right AI and the left IFG, as well as between the left PCC and the left PCC, compared to those with StD and HCs. This observation led us to identify more pronounced reductions in the FC of the DMN and SN to the IFG following the onset of MDD, which further exacerbate visual learning impairments.

This study investigated FC patterns within four major brain networks in individuals with StD through whole-brain voxel-wise analyses and evaluated their association with cognitive function to enhance the understanding of depression’s pathophysiology. This study is characterized by several strengths, including a large sample size of individuals with StD and a comprehensive assessment of cognitive function. However, certain limitations must be acknowledged. Firstly, the study utilized a cross-sectional design, involving data collection at a single time point, which limits the ability to draw definitive conclusions. Future research should employ a longitudinal design to address this limitation and to identify factors associated with the progression from StD to clinical depression. Secondly, a significant number of participants did not complete the MCCB. Participants were required to complete the cognitive assessment within 48 hours of initial contact, and the MCCB assessment tool approximately 60 minutes. Several participants withdrew due to personal circumstances that hindered their ability to complete the assessments within the specified timeframe. To investigate the issue of missing data, we conducted a comparative analysis of demographic and symptom variables between completers and non-completers within the StD and MDD groups. Our findings revealed no significant demographic or clinical differences between these groups. Consequently, the data do not substantiate the hypothesis that cognitive impairment or increased severity disproportionately elevates the likelihood of dropout. Thirdly, the alignment of diagnostic groups with specific recruitment settings – university students for the StD group and unmedicated MDD patients from hospital settings – introduces contextual confounds that have not been modeled. These confounds encompass systematic differences in socioeconomic status (SES), severity influenced by hospitalization, and unmeasured lifestyle factors, such as sleep architecture and substance use patterns. These factors may independently influence resting-state FC and performance on the MCCB, thereby potentially confounding group comparisons. Furthermore, given the exploratory nature of the correlation analyses, we did not apply a Bonferroni correction to account for multiple comparisons, which introduces the possibility of multiple comparison errors. Therefore, our findings should be considered preliminary and require validation before definitive conclusions can be established.

## Conclusions

This study investigated the FC patterns within the four major brain networks and their associations with cognitive dysfunction in StD, compared to MDD and HCs. Both StD and MDD may exhibit similar dysfunctions in the FC patterns of the SN and AN, which could influence the onset and progression of depressive symptoms. Abnormal connectivity within the DMN may be associated with the onset and recurrence of major depression. Deficits in social cognition, initially observed in individuals with StD, are associated with abnormal SN connectivity. Although individuals with StD are at an increased risk of developing MDD, not all will progress to MDD. Longitudinal studies are necessary to identify potential factors that predict the transition from StD to MDD.

## Supporting information

10.1017/S0033291725102687.sm001 Zhong et al. supplementary material Zhong et al. supplementary material

## Data Availability

The data that support the findings of this study are available from the corresponding author upon reasonable request.

## Supplementary material

The supplementary material for this article can be found at http://doi.org/10.1017/S0033291725102687.
