# Supplementary material for: Shared and distinct alterations in brain connectivity and cognitive function in subthreshold and major depression
Source: Psychol Med. 2026 Mar 9;56:e69. doi: 10.1017/S0033291725102687 (PMC12973248; doi:10.1017/S0033291725102687)
Supplement: Zhong et al. supplementary material [file S0033291725102687sup001.docx]

***Supplementary Materials***

**Shared and Distinct Alterations in Brain Connectivity and Cognitive Function in Subthreshold and Major Depression**

**
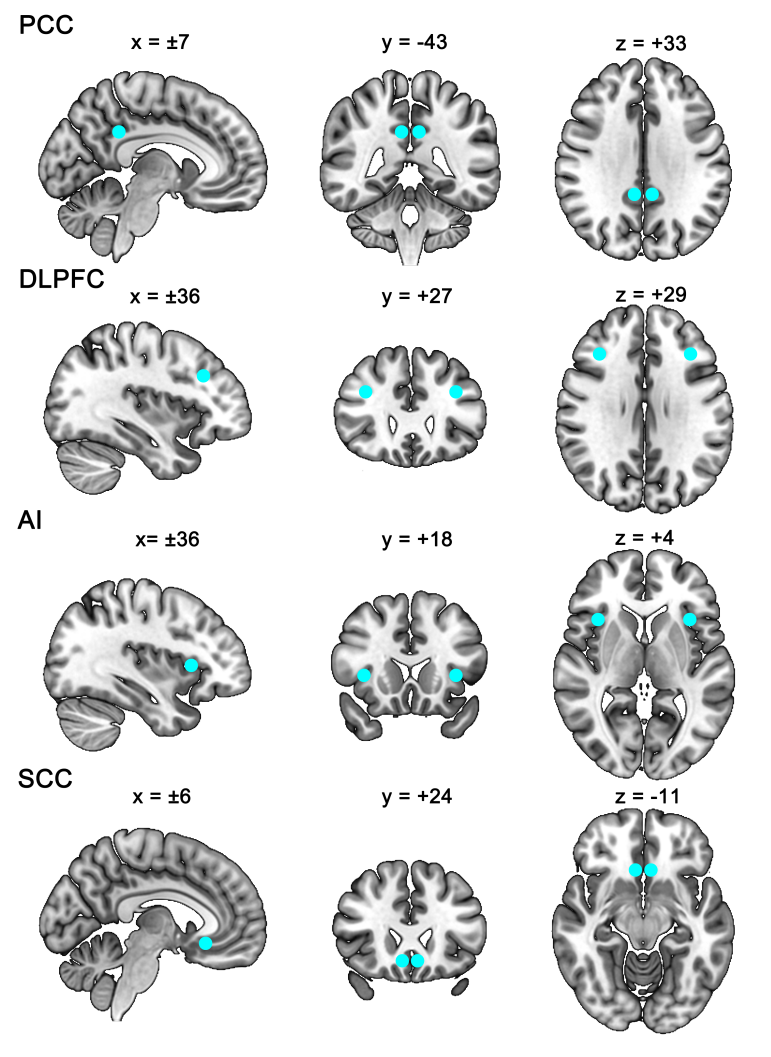
**

**Fig. S1**. Eight seeds for the four networks in the bilateral hemisphere. PCC, posterior cingulate cortex; DLPFC, dorsolateral prefrontal cortex; AI, anterior insula; SCC, subcallosal cingulate cortex.


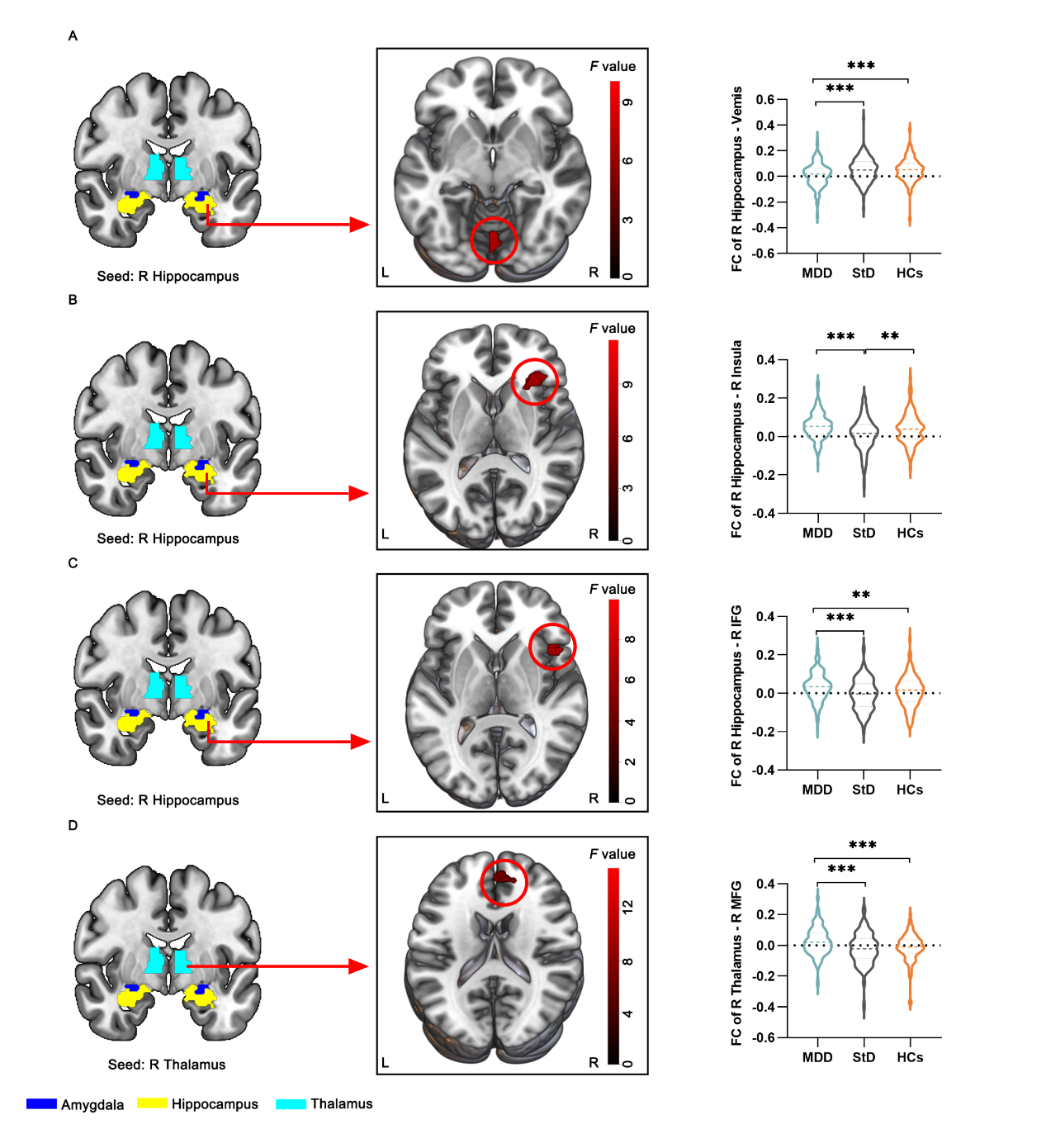


**Fig. S2** The significant FC differences among the three groups for the seeds of bilateral amygdala, hippocampus and thalamus (voxel *p* < 0.001, cluster *p* < 0.05, GRF corrected). The color bar indicates the F values from One-Way ANOVA analyses. *, *p*＜0.05 significant; **, *p*＜0.01 significant; ***, *p*＜0.001 significant; StD, subthreshold depression; MDD, major depressive disorder; HCs, healthy controls; FC, functional connectivity; GRF, Gaussian random field; IFG, inferior frontal gyrus; MFG, medial frontal gyrus; L / R, left / right hemisphere.


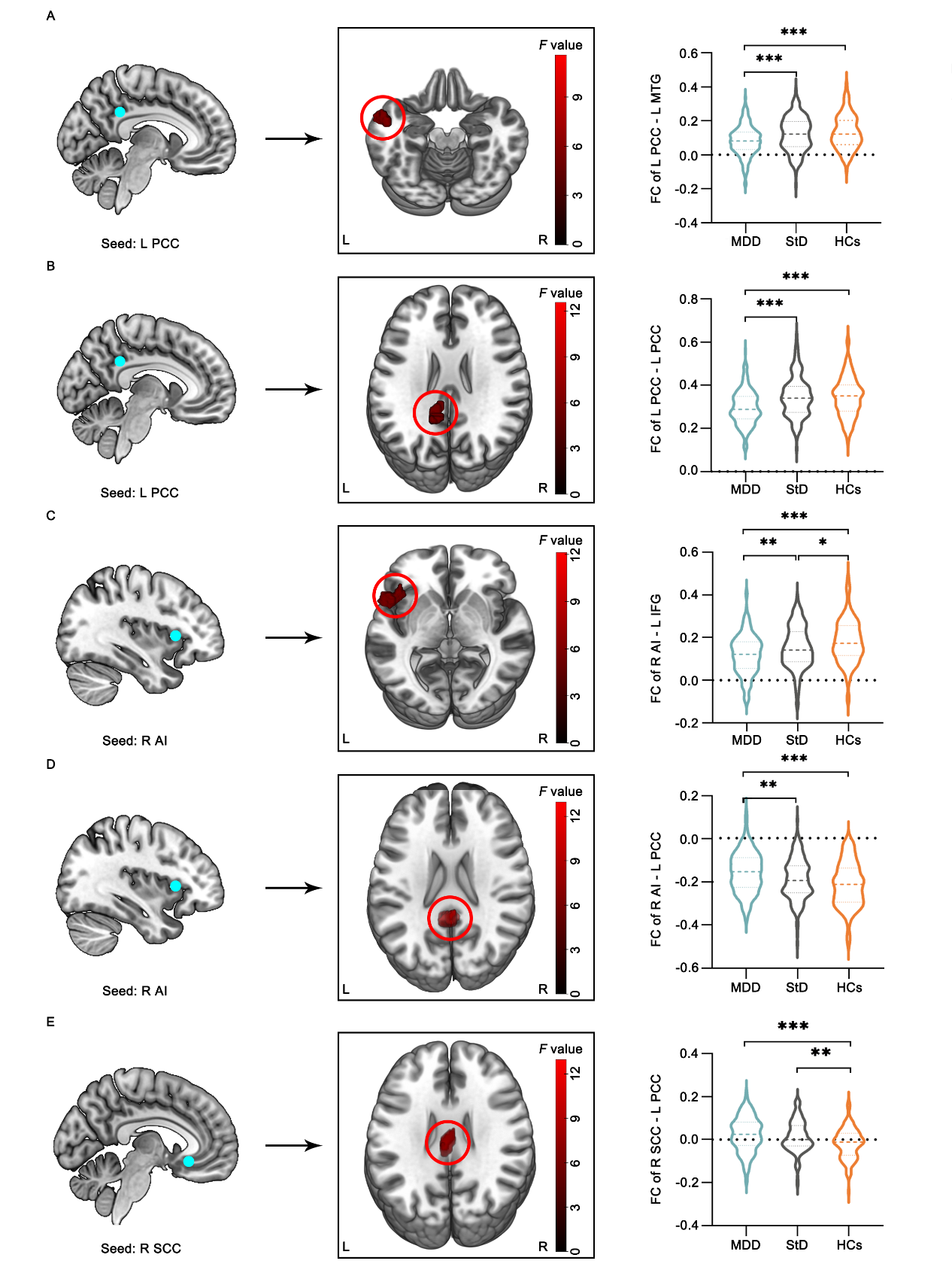
**Fig. S3** The significant FC differences among the three groups for the seeds (voxel *p* < 0.005, cluster *p* < 0.0125, GRF corrected). The color bar indicates the F values from One-Way ANOVA analyses. *, *p*＜0.05 significant; **, *p*＜0.01 significant; ***, *p*＜0.001 significant; StD, subthreshold depression; MDD, major depressive disorder; HCs, healthy controls; FC, functional connectivity; GRF, Gaussian random field; PCC, posterior cingulate cortex; MTG, middle temporal gyrus; AI, anterior insula; IFG, inferior frontal gyrus; SCC, subcallosal cingulate cortex; L / R, left / right hemisphere.


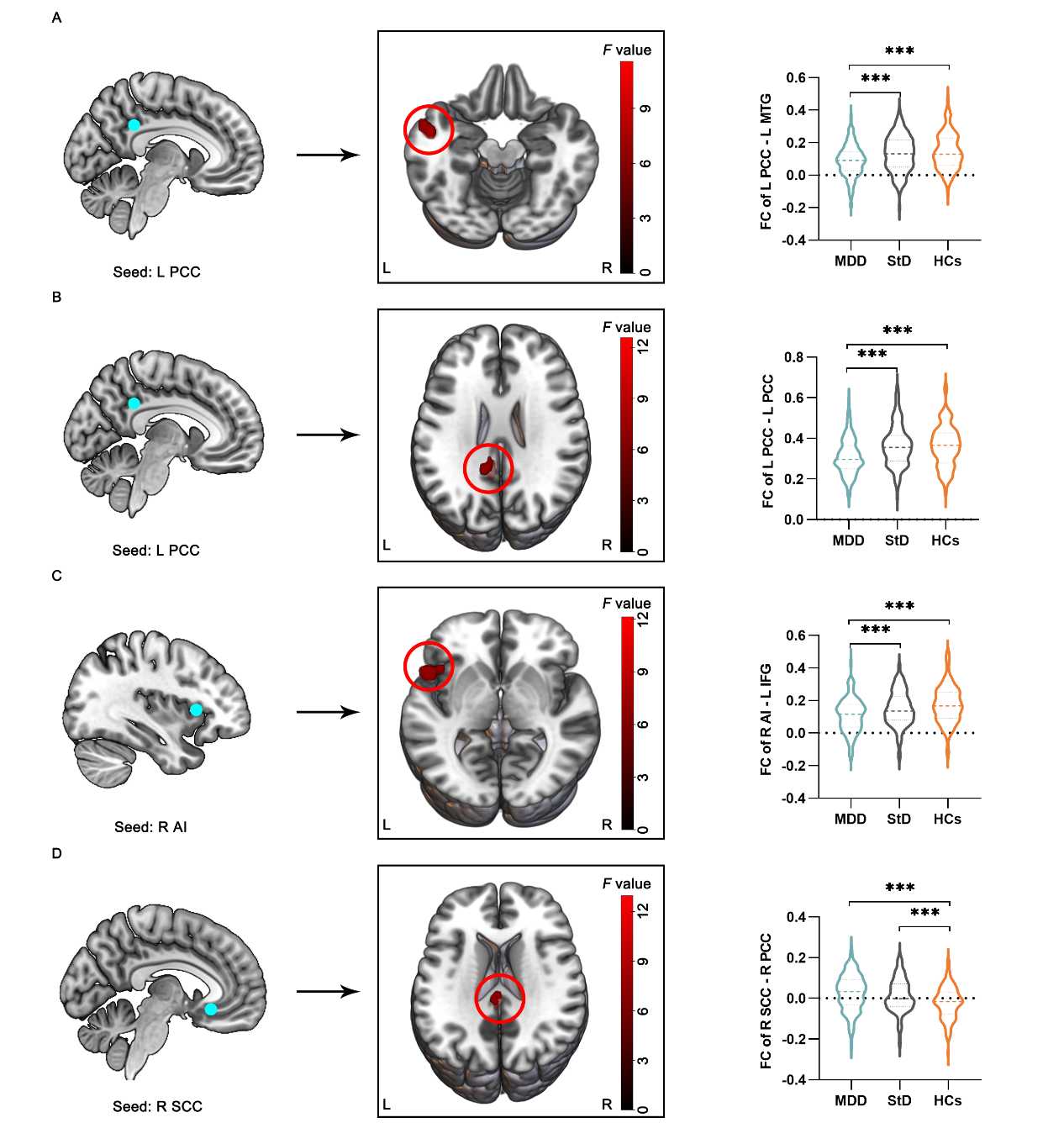


**Fig. S4** The significant FC differences among the three groups for the seeds (voxel *p* < 0.001, cluster p < 0.05, GRF corrected). The color bar indicates the F values from One-Way ANOVA analyses. *, *p*＜0.05 significant; **, *p*＜0.01 significant; ***, *p*＜0.001 significant; StD, subthreshold depression; MDD, major depressive disorder; HCs, healthy controls; FC, functional connectivity; GRF, Gaussian random field; PCC, posterior cingulate cortex; AI, anterior insula; IFG, inferior frontal gyrus; SCC, subcallosal cingulate cortex; L / R, left / right hemisphere.

**
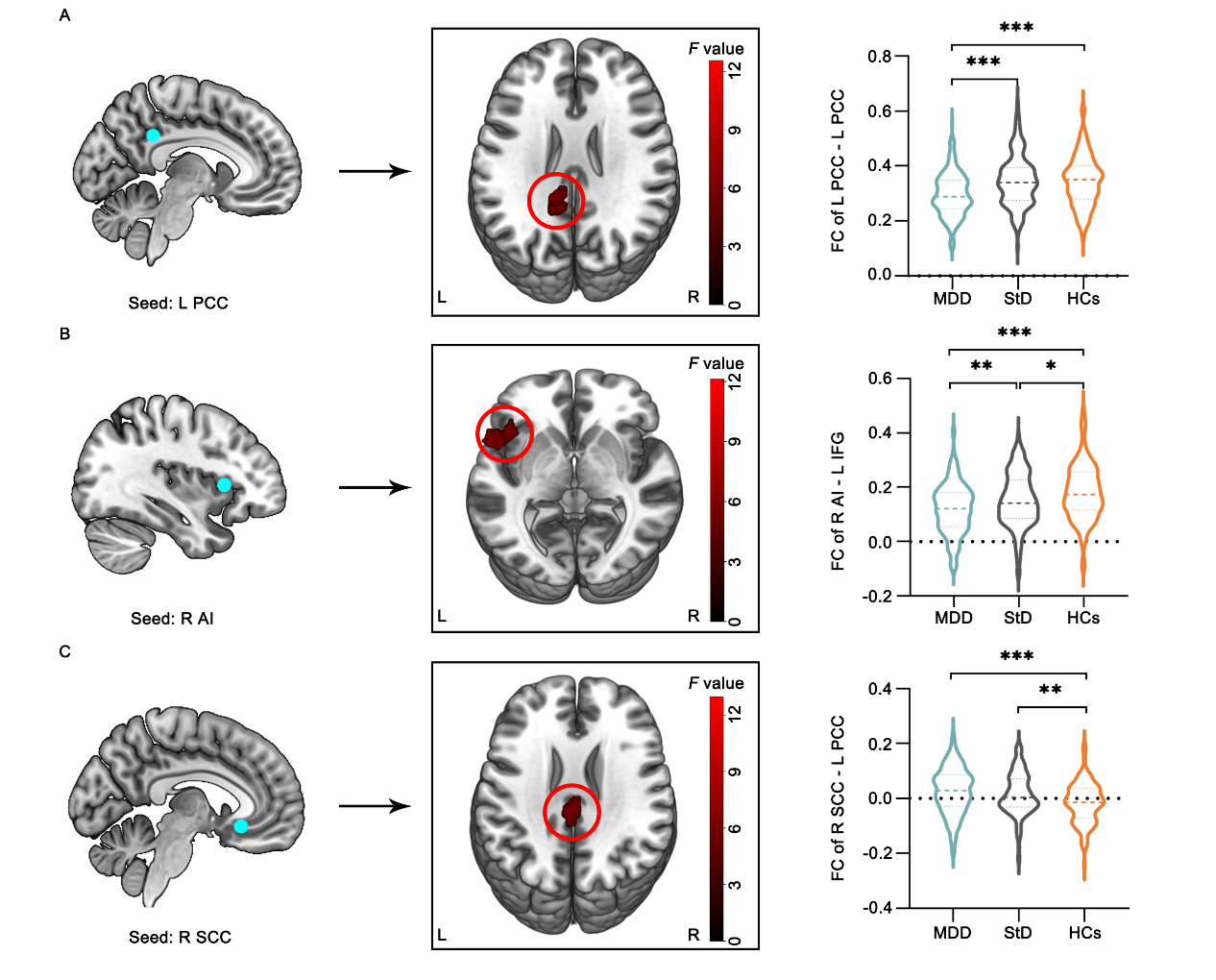
**

**Fig. S5** The significant FC differences among the three groups for the seeds (voxel *p* < 0.005, cluster *p* < 0.00625, GRF corrected). The color bar indicates the F values from One-Way ANOVA analyses. *, *p*＜0.05 significant; **, *p*＜0.01 significant; ***, *p*＜0.001 significant; StD, subthreshold depression; MDD, major depressive disorder; HCs, healthy controls; FC, functional connectivity; GRF, Gaussian random field; PCC, posterior cingulate cortex; AI, anterior insula; IFG, inferior frontal gyrus; SCC, subcallosal cingulate cortex; L / R, left / right hemisphere.


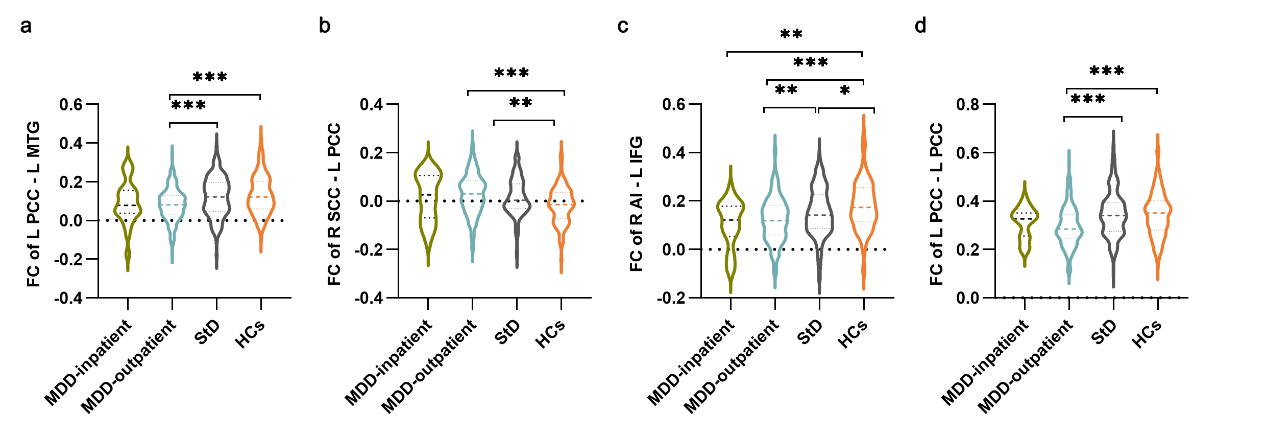


**Fig. S6** The significant FC differences among the four groups for the seeds. *, *p*＜0.05 significant; **, *p*＜0.01 significant; ***, *p*＜0.001 significant; StD, subthreshold depression; MDD, major depressive disorder; HCs, healthy controls; FC, functional connectivity; GRF, Gaussian random field; PCC, posterior cingulate cortex; MTG, middle temporal gyrus; AI, anterior insula; IFG, inferior frontal gyrus; SCC, subcallosal cingulate cortex; L / R, left / right hemisphere.


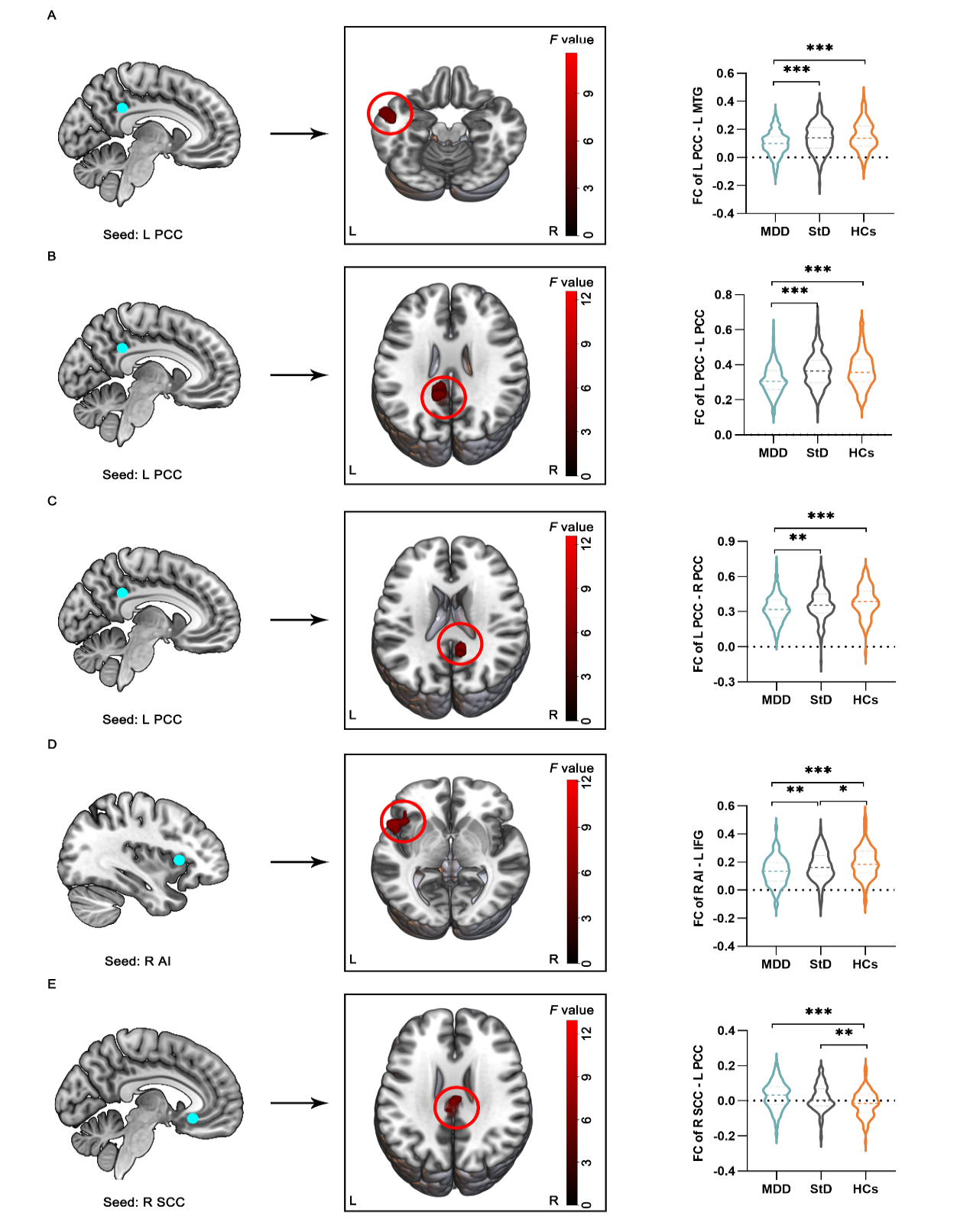


**Fig. S7** The significant FC differences among the three groups for the seeds (voxel *p* < 0.005, cluster *p* < 0.0125, GRF corrected). The color bar indicates the F values from One-Way ANOVA analyses. *, *p*＜0.05 significant; **, *p*＜0.01 significant; ***, *p*＜0.001 significant; StD, subthreshold depression; MDD, major depressive disorder; HCs, healthy controls; FC, functional connectivity; GRF, Gaussian random field; BA, Brodmann Area; MNI, Montreal Neurological coordinate; PCC, posterior cingulate cortex; MTG, middle temporal gyrus; AI, anterior insula; IFG, inferior frontal gyrus; SCC, subcallosal cingulate cortex; L / R, left / right hemisphere.

**
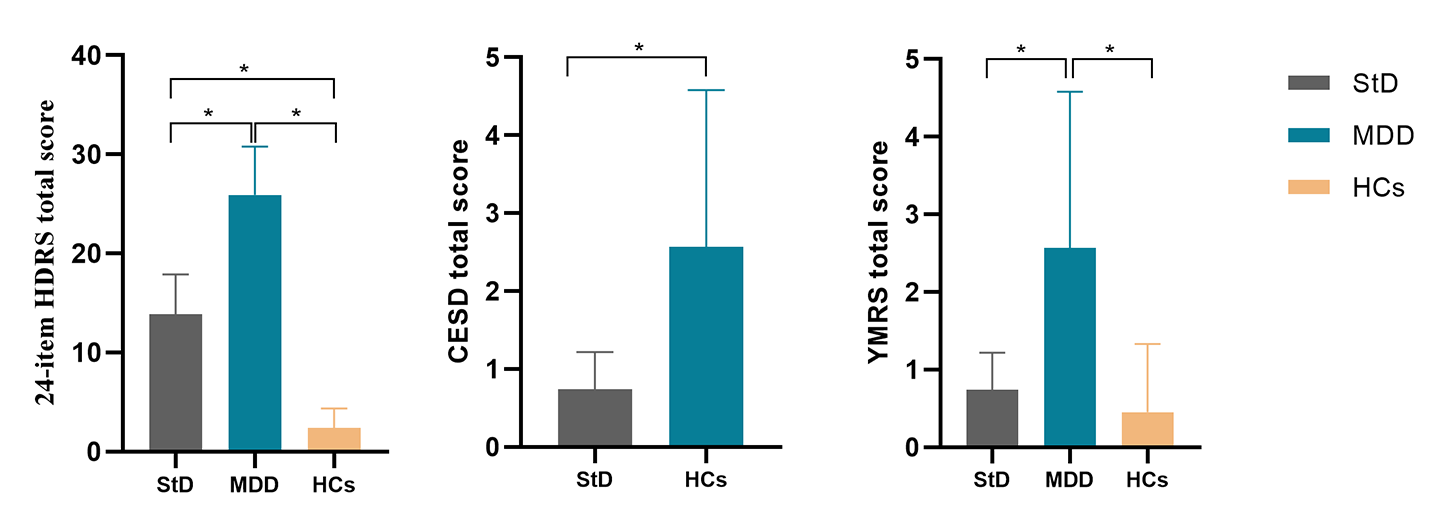
**

**Fig. S8**. Comparison of clinical scales among StD, MDD and HCs. StD, subthreshold depression; MDD, major depressive disorder; HCs, healthy controls; HDRS, Hamilton Depression Rating Scale; CESD, center for epidemiological survey depression scale; YMRS, Young Mania Rating Scale; ^*^, *p* ＜0.05 significant.


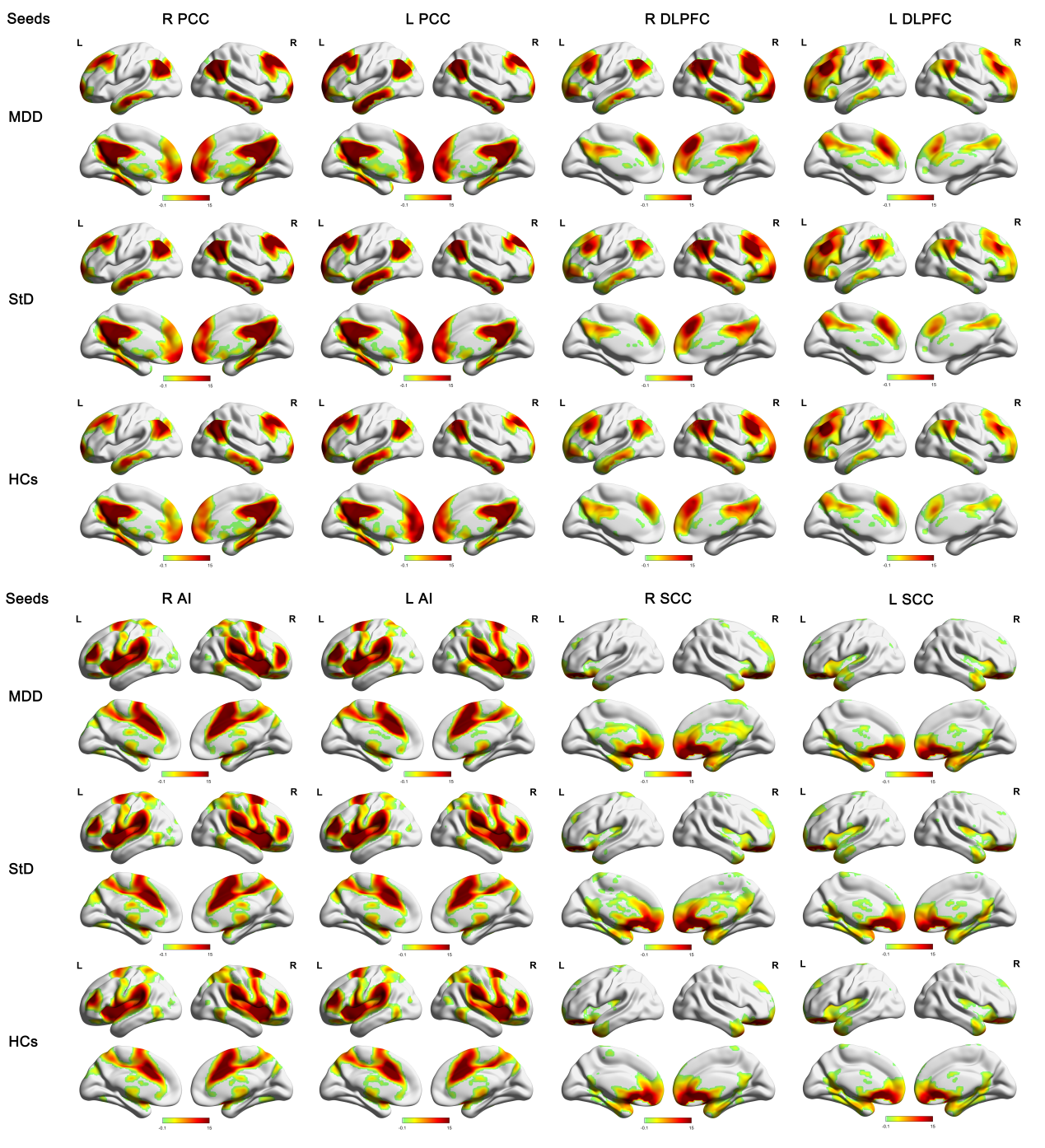


**Fig. S9**. The FC patterns of the seeds in the MDD, StD and HCs group. Shades of red represent increased FC regions. FC, functional connectivity; MDD, major depressive disorder; StD, subthreshold depression; HCs, healthy controls; PCC, posterior cingulate cortex; DLPFC , dorsolateral prefrontal cortex ; AI, anterior insula; SCC, subcallosal cingulate cortex; L (R), left (right) hemisphere.

**Table S1** Demographics and clinical data of participants included in cognitive function analysis [mean (SD)]

|  | **MDD group** | **StD group** | **HCs** | ***F*, *X^2^*, *Z*, *t*** | ***p value*** | ***p value ^e^*** |
| --- | --- | --- | --- | --- | --- | --- |
| Number of subjects | 140 | 124 | 90 | - | ***-*** |  |
| Age (year) | 22.64 (3.88) | 21.89 (2.48) | 21.88 (3.04) | 2.291 | 0.103 ^a^ |  |
| Age range (year) | 18-45 | 18-45 | 18-45 | - | - |  |
| Gender (male/female) | 51/89 | 46/78 | 38/52 | 0.867 | 0.648 ^b^ |  |
| Education (year) | 14.91 (1.88) | 15.43 (2.22) | 15.31 (2.40) | 2.123 | 0.121 ^a*^ |  |
| 24-item HDRS score | 25.66 (4.67) | 14.04 (3.95) | 2.35 (1.91) | 305.985 | <0.001 ^c*^ | MDD > StD > HCs ^e^ |
| CESD score | - | 26.47 (7.12) | 5.53 (4.22) | 29.58 | <0.001 ^d*^ | StD > HCs ^e^ |
| YMRS score | 2.27 (1.89) | 0.64 (0.44) | 0.42 (0.83) | 31.884 | <0.001 ^c*^ | MDD < StD, HCs ^e^ |

MDD, major depressive disorder; StD, subthreshold depression; HCs, healthy controls; HDRS, Hamilton Depression Rating Scale; CESD, center for epidemiological survey depression scale; YMRS, Young Mania Rating Scale; ^a^, One-Way ANOVA analyses; ^b^, X^2^ test;^c^, Kruskal-Wallis test; ^d^, Independent-sample *t*-test; ^e^, Bonferroni post hoc test; *^*^*, *p*＜0.05 significant.

Table S1 showed the demographic and clinical data of participants included in cognitive function analysis. Significantly, there were no notable variations in age, gender, or years of education among the MDD, StD group and HCs group (*p* > 0.05). Both MDD and StD group showed significantly higher 24-item HDRS-24 than HCs (both *p* < 0.001), and the 24-item HDRS-24 score in MDD group were higher than that in StD group (*p* < 0.001). In addition, MDD showed significantly higher YMRS score than StD and HCs (both *p* < 0.001).

**Table S2** Comparisons of cognitive function indices among StD, MDD and HCs group [mean (SD)]

|  | **StD group (n=124)** | **MDD group (n=140)** | **HCs**  **(n=90)** | ***F*** | ***p value*** | ***p value ^e^*** |
| --- | --- | --- | --- | --- | --- | --- |
| Speed of processing | 48.60 (8.42) | 46.14 (11.01) | 50.77 (8.38) | 6.267 | 0.002 ^f*^ | MDD < HCs ^e^ |
| Attention/vigilance | 49.24 (8.30) | 45.47 (9.06) | 50.13 (6.70) | 9.878 | < 0.001 ^f*^ | MDD < StD, HCs ^e^ |
| Working memory | 47.52 (9.14) | 45.54 (10.77) | 50.02 (9.75) | 6.246 | 0.002 ^f*^ | MDD < HCs ^e^ |
| Verbal learning | 49.29 (7.14) | 44.27 (10.28) | 50.11 (8.45) | 15.992 | < 0.001 ^f*^ | MDD < StD, HCs ^e^ |
| Visual learning | 53.01 (5.76) | 46.99 (9.36) | 53.40 (6.13) | 28.550 | < 0.001 ^f*^ | MDD < StD, HCs ^e^ |
| Reasoning/problem-solving | 50.61 (9.85) | 48.14 (10.60) | 50.77 (10.12) | 2.912 | 0.056 ^f^ |  |
| Social cognition | 48.06 (11.88) | 46.55 (12.19) | 56.05 (10.91) | 18.823 | < 0.001 ^f*^ | MDD, StD < HCs ^e^ |
| Composite | 49.26 (6.19) | 43.78 (10.43) | 52.44 (6.82) | 31.863 | < 0.001 ^f*^ | MDD < StD < HCs ^e^ |

StD, subthreshold depression; MDD, major depressive disorder; HCs, healthy controls; ^f^, One-Way ANOVA with years of education as covariate; ^e^, Bonferroni post hoc test;*^*^*, *p*＜0.05 significant.

**Table S3** Comparisons of cognitive function indices among StD, MDD (outpatient) and HCs group [mean (SD)]

|  | **StD group (n=124)** | **MDD group (n=124)** | **HCs**  **(n=90)** | ***F*** | ***p value*** | ***p value ^e^*** |
| --- | --- | --- | --- | --- | --- | --- |
| Speed of processing | 48.60 (8.42) | 46.26 (11.15) | 50.77 (8.38) | 11.630 | 0.003 ^c*^ | MDD < HCs ^e^ |
| Attention/vigilance | 49.24 (8.30) | 45.99 (8.74) | 50.13 (6.70) | 13.735 | < 0.001 ^c*^ | MDD < StD, HCs ^e^ |
| Working memory | 47.52 (9.14) | 45.27 (10.69) | 50.02 (9.75) | 6.048 | 0.003 ^a*^ | MDD < HCs ^e^ |
| Verbal learning | 49.29 (7.15) | 44.37 (10.55) | 50.11 (8.45) | 21.091 | < 0.001 ^c*^ | MDD < StD, HCs ^e^ |
| Visual learning | 53.01 (5.76) | 46.88 (9.46) | 53.40 (6.13) | 41.409 | < 0.001 ^c*^ | MDD < StD, HCs ^e^ |
| Reasoning/problem-solving | 50.61 (9.85) | 48.43 (10.54) | 50.77 (10.12) | 1.917 | 0.149 ^a^ |  |
| Social cognition | 48.06 (11.88) | 46.75 (11.92) | 56.04 (10.91) | 18.405 | < 0.001 ^a*^ | MDD, StD < HCs ^e^ |
| Composite | 49.26 (6.19) | 43.97 (10.42) | 52.44 (6.82) | 43.363 | < 0.001 ^c*^ | MDD < StD < HCs ^e^ |

StD, subthreshold depression; MDD, major depressive disorder; HCs, healthy controls; ^a^, One-Way ANOVA; ^c^, Kruskal-Wallis test; ^e^, Bonferroni post hoc test;*^*^*, *p*＜0.05 significant.

**Table S4** Demographics and clinical data between completers and non-completers in StD, MDD and HCs [mean (SD)].

|  | **Completers** | **Non-completers** | ***t, X^2^*, *Z*** | ***p value*** |
| --- | --- | --- | --- | --- |
| **StD** |  |  |  |  |
| Number of subjects | 124 | 29 | - | ***-*** |
| Age (year) | 21.89 (2.48) | 23.07 (4.09) | -1.494 | 0.145 ^a^ |
| Age range (year) | 18-45 | 18-45 | - | - |
| Gender (male/female) | 46/78 | 9/20 | 0.375 | 0.540 ^b^ |
| Education (year) | 15.43 (2.22) | 16.31 (3.31) | -1.351 | 0.186 ^a^ |
| 24-item HDRS score | 14.04 (3.95) | 12.74 (4.07) | 1.334 | 0.185 ^c^ |
| CESD score | 26.47 (7.12) | 25.35 (6.16) | 0.918 | 0.360 ^c^ |
| YMRS score | 0.64 (0.44) | 0.53 (0.24) | 0.793 | 0.372 ^c^ |
| **MDD** |  |  |  |  |
| Number of subjects | 140 | 48 | - | - |
| Age (year) | 22.64 (3.88) | 22.96 (5.09) | -0.457 | 0.648 ^a^ |
| Age range (year) | 18-45 | 18-45 | - | - |
| Gender (male/female) | 51/89 | 18/30 | 0.018 | 0.894 ^b^ |
| Education (year) | 14.91 (1.88) | 14.21 (2.41) | 3.314 | 0.141 ^a^ |
| 24-item HDRS score | 25.66 (4.67) | 26.68 (5.32) | -1.263 | 0.208 ^c^ |
| YMRS score | 2.27 (1.89) | 2.01 (1.59) | 0.234 | 0.548 ^c^ |
| **HCs** |  |  |  |  |
| Number of subjects | 90 | 20 | - | - |
| Age (year) | 21.88 (3.04) | 24.65 (2.98) | -3.704 | < 0.001 ^a*^ |
| Age range (year) | 18-45 | 18-45 | - | - |
| Gender (male/female) | 38/52 | 10/10 | 0.402 | 0.526 ^b^ |
| Education (year) | 15.31 (2.40) | 17.80 (2.44) | -4.187 | < 0.001 ^a*^ |
| 24-item HDRS score | 2.35 (1.91) | 2.53 (2.40) | -0.336 | 0.738 ^c^ |
| CESD score | 5.53 (4.22) | 4.75 (4.08) | 0.911 | 0.364 ^c^ |
| YMRS score | 0.42 (0.83) | 0.34 (0.79) | 0.412 | 0.687 ^c^ |

StD, subthreshold depression; MDD, major depressive disorder; HCs, healthy controls; HDRS, Hamilton Depression Rating Scale; CESD, center for epidemiological survey depression scale; YMRS, Young Mania Rating Scale; ^a^, independent two-sample t-tests; ^b^, X^2^ test; ^c^, Mann-Whitney U test; *^*^*, *p*＜0.05 significant.
